# Measurement of Baseline and Orientation between Distributed Aerospace Platforms

**DOI:** 10.1155/2013/985601

**Published:** 2013-06-04

**Authors:** Wen-Qin Wang

**Affiliations:** The School of Communication and Information Engineering, University of Electronic Science and Technology of China (UESTC), Chengdu 611731, China

## Abstract

Distributed platforms play an important role in aerospace remote sensing, radar navigation, and wireless communication applications. However, besides the requirement of high accurate time and frequency synchronization for coherent signal processing, the baseline between the transmitting platform and receiving platform and the orientation of platform towards each other during data recording must be measured in real time. In this paper, we propose an improved pulsed duplex microwave ranging approach, which allows determining the spatial baseline and orientation between distributed aerospace platforms by the proposed high-precision time-interval estimation method. This approach is novel in the sense that it cancels the effect of oscillator frequency synchronization errors due to separate oscillators that are used in the platforms. Several performance specifications are also discussed. The effectiveness of the approach is verified by simulation results.

## 1. Introduction

Distributed aerospace platforms play a more and more important role in aerospace remote sensing, radar navigation, and wireless communication applications [1–3]. One representative application example is the distributed synthetic aperture radar (SAR) remote sensing [4–6], especially the distributed interferometry SAR (InSAR) remote sensing which is an imaging technique for measuring the topography of surface, along with its changes over time [7]. They are of fundamental importance for a broad range of commercial and scientific applications [8]. For example, many geoscience areas, like hydrology, glaciology, forestry, geology, oceanography, and land environment, require precise and up-to-date information about the earth's surface and its topography.

Conventional repeat-pass InSAR may suffer from temporal decorrelation and atmospheric distortions. Such disadvantages may be avoided by using distributed InSAR, where separate transmitter and receiver are used. Distributed InSAR enables a flexible imaging geometry with large baselines, thereby increasing significantly the interferometric performance [9–11]. Recently, advances in distributed InSAR techniques have addressed some of the limitations in conventional InSAR systems and subsequently have opened many new remote sensing applications [12–14], because it can avoid time decorrelation between the interferometric signals and improve the topographic measurement sensitivity owing to large baseline formed between the transmitter and receiver. However, besides the requirement of highly accurate time and frequency synchronization for coherent signal processing [15], one has to determine the baseline between transmitter and receiver and the orientation of platform towards each other during data recording. These parameters directly affect the achievable geometrical resolution and the geometry of the radar system.

To determine baseline and orientation for bistatic airborne radars, a method by installing 4 × 4 dedicated navigation units on the transmitter and receiver was proposed in [16]. The approach is further investigated in [17] by employing only two transmitting antennas and two receiving antennas. However, the influence of oscillator synchronization errors is not considered, and several contributions such as the influence of ionosphere or relativistic effects are also neglected. We are aware that these contributions might become significant. So this paper extends the approach to include additional factors such as oscillator frequency synchronization errors, Doppler effects, and ionosphere or relativistic effects, which are crucial for ensuring the performance of baseline and orientation estimation.

This paper concentrates on the measurement of baseline and orientation between distributed aerospace platforms for general aerospace applications, not for distributed InSAR remote sensing only. In distributed aerospace platforms, the baseline and orientation will change during the flying time and they should be estimated in real time [18], due to the unstability of the separate platforms. To reach this aim, this paper proposes an improved pulsed duplex microwave ranging technique to determine the spatial baseline and orientation between distributed aerospace platforms.

The remaining sections of this paper are organized as follows. To adaptively estimate the change of spatial baseline and orientation between the transmitting platform and receiving platform, Section 2 proposes a high precision microwave ranging approach.  Section 3 presents a high-precision time-interval estimation algorithm. Next, the baseline and orientation estimation performance is analyzed in Section 4. Finally, this paper is concluded in Section 5.

## 2. Estimation of Baseline and Orientation

In many applications, the baseline between distributed aerospace platforms and the orientation towards each other must be measured in a high precision manner during data recording. Up to now, these parameters are usually determined using independent global navigation satellite system (GNSS)—like the GPS—and differential GPS systems (DGPS) receivers on each platform. The achievable accuracy of a DGPS in real time is about 10 cm and several millimeters after postprocessing. However, much higher accuracy is required for some specific applications; for example, an 0.3 mm accuracy is needed for an X-band distributed InSAR system [16].

To adaptively determine the baseline between the transmitting platform and receiving platform and the orientation towards each other, we propose a pulsed duplex microwave ranging method. In this method, the transmitting navigation unit generates a signal from one stable local oscillator and splits it into three individual navigation signals. Each of these signals is modulated and transmitted over separate antennas. These navigation antennas are pointing towards the receiving platform. In this way, the baseline and orientation between the platforms can be adaptively measured.

**Figure 1:**
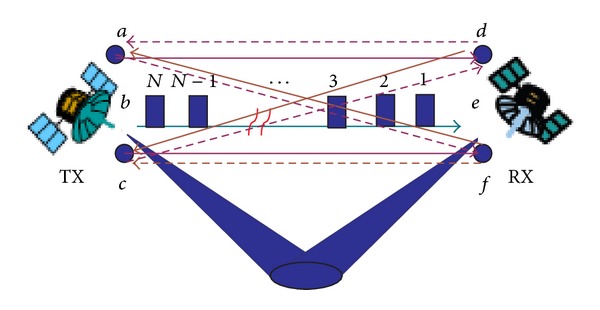
Two aerospace platforms and possible combination of navigation signals.

Figure 1 shows a configuration of 3 × 3 two-way ranging technique; the navigation signal is modulated by pseudo-random noise (PN) codes. These PN codes are chosen from a set of gold codes and are unique for each navigation signal; hence, the correlation between any pair of codes is very low. This allows us to use the same carrier frequency for all the navigation signals, so that the effects of oscillator frequency synchronization errors can be canceled out. Moreover, some additional information such as the position of the antennas and the position of the platform is also modulated with these signals. At time *T*_TX_, the three navigation signals are transmitted from the TX using separate antennas and therefore travel on different paths towards the RX. Similarly, there are three navigation antennas installed in the RX platform. They are received by the RX antenna *d* at times *T*_RX*ad*_, *T*_RX*bd*_, and *T*_RX*cd*_, respectively. For the other two RX antennas, there are similar results. After frame synchronization, the messages can then be decoded and used for subsequent range calculations. The pseudorange between the TX navigation antennas and each RX navigation antenna can be determined by the time of the signal propagations.

 But the measured time may be biased due to the lack of time synchronization between the clocks inside the TX and RX platforms. Additionally, the time may be biased due to the effects of ionosphere and receiver noise. Hence, for the RX antenna *d*, the measured time equation can be expressed as

(1) $$\begin{array}{c} \Delta T_{ad}=\left(T_{\text{RX}ad}-T_{\text{TX}}\right)+\Delta T_{\text{syn}}+\Delta t_{\text{ion}}+\Delta t_{\text{noi}},\\ \Delta T_{bd}=\left(T_{\text{RX}bd}-T_{\text{TX}ab}\right)+\Delta T_{\text{syn}}+\Delta t_{\text{ion}}+\Delta t_{\text{noi}},\\ \Delta T_{cd}=\left(T_{\text{RX}cd}-T_{\text{TX}ab}\right)+\Delta T_{\text{syn}}+\Delta t_{\text{ion}}+\Delta t_{\text{noi}}, \end{array}$$

where Δ*T*_syn_, Δ*t*_ion_, and Δ*t*_noi_ denote the time offsets caused by time synchronization errors, ionosphere effect, and receiver noise, respectively.

As the effect of ionosphere can be compensated to satisfactory accuracy by using experiential radio propagation models, for example, [19]

(2) $$\begin{array}{c} \Delta t_{\text{ion}}=\frac{A}{c_{0}\cdot f_{0}^{2}}, \end{array}$$

where *A* is one constant, and *c*_0_ and *f*_0_ are the speed of light and carrier frequency, respectively. Equation (1) can then be further simplified into

(3) $$\begin{array}{c} \Delta T_{ad}^{\prime }=\left(T_{\text{RX}ad}-T_{\text{TX}}\right)+\Delta T_{\text{syn}}+\Delta t_{\text{noi}},\\ \Delta T_{bd}^{\prime }=\left(T_{\text{RX}bd}-T_{\text{TX}}\right)+\Delta T_{\text{syn}}+\Delta t_{\text{noi}},\\ \Delta T_{cd}^{\prime }=\left(T_{\text{RX}cd}-T_{\text{TX}}\right)+\Delta T_{\text{syn}}+\Delta t_{\text{noi}}. \end{array}$$

Similarly, for the RX antennas *e* and *f*, we also have

(4) $$\begin{array}{c} \Delta T_{ie}^{\prime }=\left(T_{\text{RX}ie}-T_{\text{TX}}\right)+\Delta T_{\text{syn}}+\Delta t_{\text{noi}}, \end{array}$$

(5) $$\begin{array}{c} \Delta T_{if}^{\prime }=\left(T_{\text{RX}if}-T_{\text{TX}}\right)+\Delta T_{\text{syn}}+\Delta t_{\text{noi}}, \end{array}$$

where *i* ∈ (*a*, *b*, *c*).

To cancel out the effect of oscillator frequency synchronization errors, three navigation signals are transmitted from the RX platform to the TX platform at time *T*_RX_. In a like manner, we can get

(6) $$\begin{array}{c} \Delta T_{ji}^{\prime }=\left(T_{\text{TX}ji}-T_{\text{RX}}\right)-\Delta T_{\text{syn}}+\Delta t_{\text{noi}}, \end{array}$$

with *j* ∈ (*d*, *e*, *f*). Note that the *T*_TX*ji*_ are defined as previously. Since

(7) $$\begin{array}{c} T_{\text{RX}ij}-T_{\text{TX}}≃T_{\text{TX}ji}-T_{\text{RX}} \end{array}$$

We can obtain the signal propagation time between the TX antennas and RX antennas:

(8) τij=τji=ΔTij′+ΔTji′2−Δtnoi≈ΔTij′+ΔTji′2,ΔTsyn=ΔTij′−ΔTji′2.

Hence, the pseudo-range between the TX navigation antennas (*i* ∈ (*a*, *b*, *c*)) and TX navigation antennas (*i* ∈ (*d*, *e*, *f*)) is the time offset *τ*_*ij*_ (or *τ*_*ji*_) multiplied by the speed of light *c*_0_ and is biased by the effect of receiver noise investigated in subsequent section:

(9) $$\begin{array}{c} R_{ij}=R_{ji}=c_{0}\cdot \tau_{ij}\approx c_{0}\cdot \frac{\Delta T_{ij}^{\prime }+\Delta T_{ji}^{\prime }}{2}. \end{array}$$

Thus, the relative distance between the TX platform and RX platform can be determined from the calculated pseudo-range. As an example, for the RX antenna *d* we can get

(10) (xi−xd)2+(yi−yd)2+(zi−zd)2=Rid2,(xi−xe)2+(yi−ye)2+(zi−ze)2=Rie2,(xi−xf)2+(yi−yf)2+(zi−zf)2=Rif2.

In this way, using the three determined potions of the navigation antennas on the RX platform (*x*_*j*_, *y*_*j*_, *z*_*j*_), the spatial baseline and orientation between both platforms can be determined. More importantly, this method can cancel the effect of oscillator frequency synchronization errors.

To obtain pseudo-range, frame synchronization is required, which generally involves two steps: PN codes acquisition and tracking. In the first stage, sliding correlator is usually used to reduce the time uncertainty between local replica PN codes and received codes. The timing uncertainty covers a region that is quantized into a finite number of cells. These cells are serially tested until it is determined that a particular cell corresponds to the alignment of the two sequences to within a fraction of chip. In codes tracking stage, a delay-locked loop (DLL) [20, 21] can be used.

**Figure 2:**
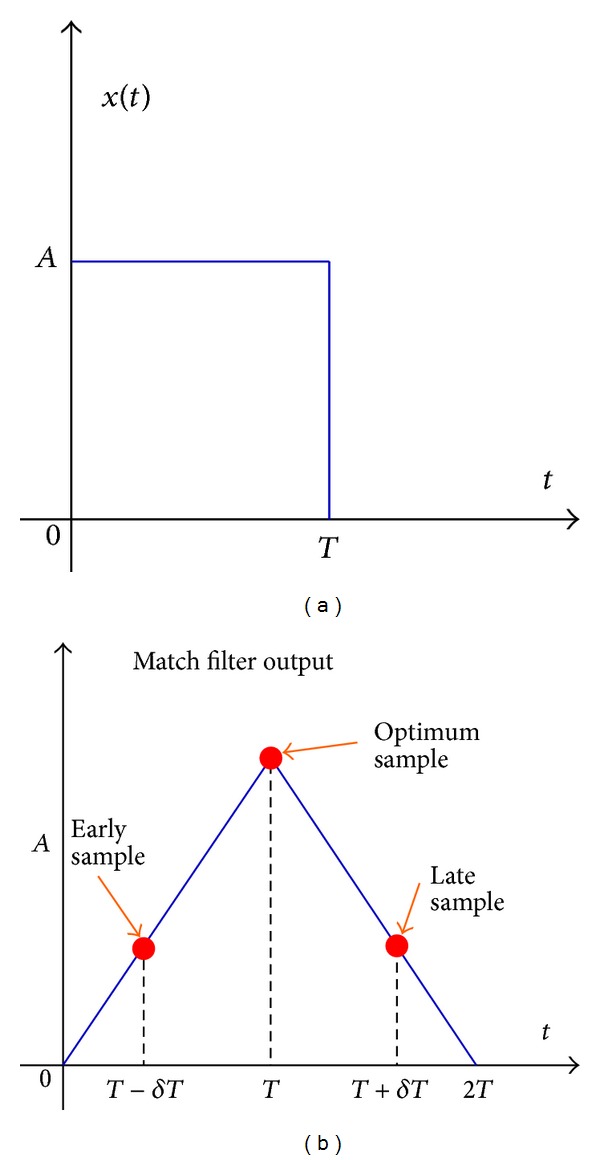
Rectangular signal pulse (a) and its matched filter output (b).

To describe this method, let us consider a rectangular pulse *x*(*t*),  0 ≤ *t* ≤ *T*, the output of a filter matched to *x*(*t*) attains its maximum value at time *t* = *T*, that is, at the peak of the correlation function, as shown in Figure 2. In the presence of noise, the identification of the peak value will be difficult. Instead of sampling the signal at peak, suppose we sample early at *t* = *T* − *δ* and late at *t* = *T* + *δ*. The absolute value of the early samples |*S*[*n*(*T* − *δ*)]| and the late samples |*S*[*n*(*T* + *δ*)]| will be smaller than the samples of the peak value |*S*[*n*(*T*)]|. As the autocorrelation function is even with respect to the optimum sampling time *t* = *T*, the absolute values of the correlation function at *t* = *T* − *δ* and *t* = *T* + *δ* are equal. Under this condition, the proper sampling time is the midpoint between *t* = *T* − *δ* and *t* = *T* + *δ*.

**Figure 3:**
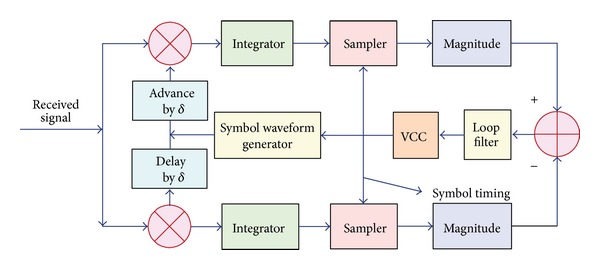
Block diagram of synchronizing tracker.

 This condition forms the basis for the tracking technique, as shown in Figure 3. The two correlators integrate over the symbol interval *T*, but one correlator starts integrating *δ* seconds early relative to the estimated optimum sampling time, and the other integrator starts integrating *δ* seconds late relative to the estimated optimum sampling time. An error signal is formed by taking the difference between the absolute values of the two correlator outputs. If the timing is off relative to the optimum sampling time, the average error signal at the output of the low-pass filter is nonzero, and the clock signal is either retarded or advanced, depending on the sign of the error. In this way, the smoothed error signal is used to drive a voltage compensation controller (VCC), whose output is the desired clock signal.

## 3. High-Precision Time-Interval Estimation

**Figure 4:**
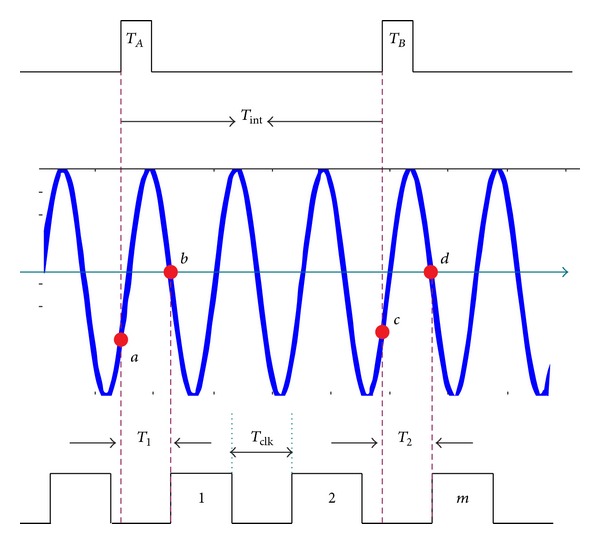
One time-interval measurement approach.

To obtain a high-precision baseline estimation, the time difference between transmission and reception must be measured. We use the high-precision time-to-phase conversion measurement technique shown in Figure 4. This technique uses the two measured signals *T*_1_ and *T*_2_ to trigger an analog-to-digital converter (DAC) to sample one sinewave signal generated from the local oscillator directly.

We start to describe this approach from a sinewave signal expressed as

(11) $$\begin{array}{c} s\left(t\right)=a_{0}\cos\left(2\pi f_{lo}t+\phi \left(t\right)+\phi_{0}\right), \end{array}$$

where *a*_0_ is the amplitude, *f*_*lo*_ is the center frequency, *ϕ*(*t*) is the phase fluctuation, and *ϕ*_0_ is the starting phase. We then have

(12) $$\begin{array}{c} s_{a}=a_{0}\cos\left(2\pi f_{lo}t_{a}+\phi \left(t_{a}\right)+\phi_{0}\right),\\ s_{b}=a_{0}\cos\left(2\pi f_{lo}\left(t_{a}+T_{1}\right)+\phi \left(t_{a}+T_{1}\right)+\phi_{0}\right),\\ s_{c}=a_{0}\cos\left(2\pi f_{lo}t_{c}+\phi \left(t_{c}\right)+\phi_{0}\right),\\ s_{d}=a_{0}\cos\left(2\pi f_{lo}\left(t_{c}+T_{2}\right)+\phi \left(t_{c}+T_{2}\right)+\phi_{0}\right), \end{array}$$

where *t*_*a*_ and *t*_*c*_ are the time at *a* and *c*, respectively, *T*_1_ is the time interval between *a* and *b*, and *T*_2_ is the time interval between *c* and *d*.

The phase terms can be further expressed as

(13) Φa=2πflota+ϕ(ta)+ϕ0=arccos(saa0)+2k1π,Φb=2πflo(ta+T1)+ϕ(ta+T1)+ϕ0=arccos(sba0)+2k1π,Φc=2πflotc+ϕ(tc)+ϕ0=arccos(sca0)+2k2π,Φd=2πflo(tc+T2)+ϕ(tc+T2)+ϕ0=arccos(sda0)+2k2π.

After the 2*π*-phase ambiguities in Φ_*a*_, Φ_*b*_, Φ_*c*_, and Φ_*d*_ being removed in a like manner that is performed in the GPS processing [22], we can get

(14) $$\begin{array}{c} T_{1}=\frac{\left(\Phi_{b}-\Phi_{a}\right)-\left(\phi \left(t_{a}+T_{1}\right)-\phi \left(t_{a}\right)\right)}{2\pi f_{lo}},\\ T_{2}=\frac{\left(\Phi_{d}-\Phi_{c}\right)-\left(\phi \left(t_{c}+T_{2}\right)-\phi \left(t_{c}\right)\right)}{2\pi f_{lo}}. \end{array}$$

Hence, the time interval between *T*_*A*_ and *T*_*B*_ signals is

(15) Tint⁡=mTclk−T1+T2=mTcl−(Φb−Φa)−(ϕ(ta+T1)−ϕ(ta))2πflo +(Φd−Φc)−(ϕ(tc+T2)−ϕ(tc))2πflo

As the parameters *T*_*clk*_, Φ_*a*_, Φ_*b*_, Φ_*c*_, Φ_*d*_, *m*, and *f*_*lo*_ are all measurable or calculable and the parameters *ϕ*(*t*_*a*_ + *T*_1_) − *ϕ*(*t*_*a*_) and *ϕ*(*t*_*c*_ + *T*_2_) − *ϕ*(*t*_*c*_) are neglectable, the time difference between *T*_1_ and *T*_2_ signals can be calculated from (15).

## 4. Design Specifications

Several factors will influence the phase of the microwave measurement link. The receiver noise determined by the signal-to-noise ratio (SNR) is of special interest; its influence on the signal phase is described by the receiver phase noise spectral density function *S*_*θ*SNR_(*f*). The hardware system error is represented by *θ*_sys_(*t*). Furthermore, the phase is sampled, which requires a later interpolation. We may choose to filter this phase with an arbitrary transfer function *H*_pf_(*f*). The link error, that is, the residual error contribution, is represented by the standard deviation (STD) *σ*_link_. This section makes an investigation on these residual influence factors such as receiver noise and Doppler effects as well as contributions known from the sampling theory.

### 4.1. Receiver Noise

 The receiver noise consists of thermal noise, and the noise collected by antenna will introduce both amplitude and phase variations to the navigation signal. Here, only the phase variations described by their spectral density function are of interest. For band-limited white noise, the spectral density function *S*_*φ*SNR_(*f*) is related to the SNR by [23]

(16) $$\begin{array}{c} S_{\varphi \text{SNR}}\left(f\right)=\frac{1}{2B_{w}\cdot \text{SNR}}, \end{array}$$

where *B*_*w*_ is the receiver noise bandwidth. Correspondingly, the receiver noise variance then is

(17) $$\begin{array}{c} \sigma_{\text{SNR}}^{2}=\frac{1}{2f_{\text{sys}}\cdot \text{SNR}}\int_{-f_{\text{sys}}/2}^{f_{\text{sys}}/2}{\left|H_{\text{pf}(\text{f})}\right|}^{2}df, \end{array}$$

where *f*_sys_ is the measurement rate. Note that here uncorrelated noise and equal SNR value are assumed for both receivers.

As an example, assuming a bistatic spaceborne InSAR system with the following parameters: *B*_*w*_ = 100 MHz, PRF  = 5000 Hz, pulse duration *τ*_*p*_ = 15 *μs*, and synthetic aperture time *T*_*s*_ = 1 s, then *S*_*φ*SNR_(*f*) is found to be smaller than −120 dBc/Hz. This noise may degrade ranging precision consequently. Generally, it is about 0.2 ~ 0.5 mm.

### 4.2. Doppler Effects

As the transmit instance of RX is delayed by *τ*_sys_ with respect to TX, the navigation signals are inherently decoupled. This offers the possibility of using a single carrier frequency. The Doppler phenomenon due to the relative velocity *v*_sat⁡_ between the TX and RX platforms may be a problem. However, the Doppler phase contribution is constant for constant relative satellite velocity *v*_sat⁡_. Only a relative satellite acceleration, that is, a time-dependent relative satellite velocity *v*_sat⁡_(*t*), will cause a measurement error because the Doppler contribution of constant relative satellite velocity *v*_sat⁡_ can be canceled out in range calculation. For severe intersatellite acceleration, a Doppler phase compensation that requires the satellite separation to be known is necessary. Fortunately, severe intersatellite acceleration is usually not existing in bistatic spaceborne InSAR systems.

### 4.3. Analog Digital Converter (ADC)

 As range estimation is processed in digital signal, analog digital converter (ADC) quantization errors may degrade the estimation precision consequently. The quantization errors result in what appears to be a white noise floor. The amplitude quantization errors *e*_*A*_ can be assumed to be totally uncorrelated and uniformly distributed within each quantization step, that is,

(18) $$\begin{array}{c} -\frac{\Delta_{A}}{2}\leq e_{A}\leq \frac{\Delta_{A}}{2}. \end{array}$$

For a D-bit ADC, the quantization step size is

(19) $$\begin{array}{c} \Delta_{A}=\frac{1}{2^{D-1}}. \end{array}$$

Then the amplitude error power is [24]

(20) $$\begin{array}{c} E\left(e_{A}^{2}\right)=\frac{1}{\Delta_{A}}\int_{-\Delta_{A}/2}^{\Delta_{A}/2}e_{A}^{2}de_{A}=\frac{\Delta_{A}^{2}}{12}. \end{array}$$

Accordingly, the noise variance is

(21) $$\begin{array}{c} \sigma =\sqrt{\frac{\Delta_{A}^{2}}{12}}=\frac{\Delta_{A}}{2\sqrt{3}}. \end{array}$$

As the signal power is

(22) $$\begin{array}{c} S={\left(2^{D}\Delta_{A}\right)}^{2}, \end{array}$$

hence the noise-signal-ratio is

(23) $$\begin{array}{c} \frac{N}{S}=10\log\frac{\sigma^{2}}{S}=20\log\frac{\Delta_{A}/2\sqrt{3}}{2^{D}\Delta_{A}}. \end{array}$$

Denoting the sampling noise bandwidth as *B*_*n*_, we then have

(24) $$\begin{array}{c} S_{\varphi_{AD}}\left(f\right)=\frac{N}{S}\cdot \frac{1}{B_{n}}=20\log\frac{\Delta_{A}/2\sqrt{3}}{2^{D}\Delta_{A}}-20\log\left(B_{n}\right). \end{array}$$

As an example, assuming the quantization bits are 12 bits and sampling rate is 300 MHz, then *S*_*φ*_*AD*__(*f*) is found to be −167.77 dBc/Hz. Correspondingly, the phase errors caused by ADC can be modeled as [25]:

(25) $$\begin{array}{c} \delta \varphi_{\max}=\tan^{-1}\left[\frac{1}{2^{D}-1}\right]. \end{array}$$

From Table 1, we can conclude that ADC has neglectable effects on the performance of range estimation.

### 4.4. Other Possible Factors

Practically, the transmitted navigation signal (from the signal generator through frequency conversion, amplification, and transmission by the navigation antenna) has some unwanted phase characteristics. Sources of unwanted errors include nonlinearity in amplifiers, antenna, link path, frequency dependent phase effects in filters and waveguide dispersion. The phase of the hardware system, dominated by active and passive radar radio frequency (RF) components, will change within the duration of data collection. Concerning the performance of the navigation link, all the contributions from components common to the TX and RX path will cancel out, due to two-day operation. In [26], an exemplary measured phase variation in a two-way synchronization link is about 0.39°. This effect is small and can be ignored.

### 4.5. DLL Ranging Estimation

**Figure 5:**
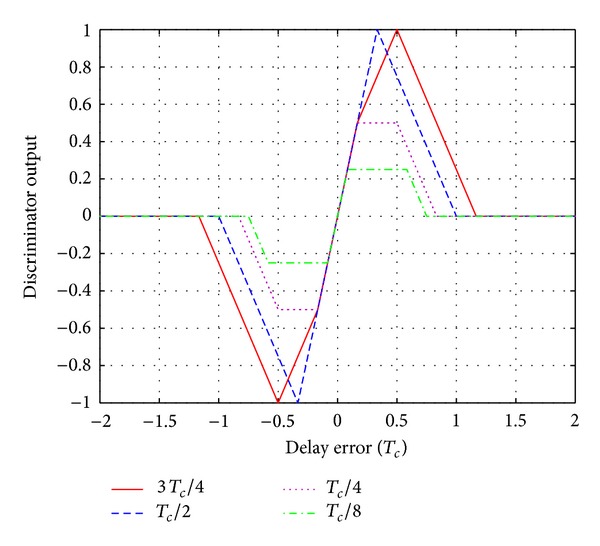
Characteristics of the discriminator.

**Figure 6:**
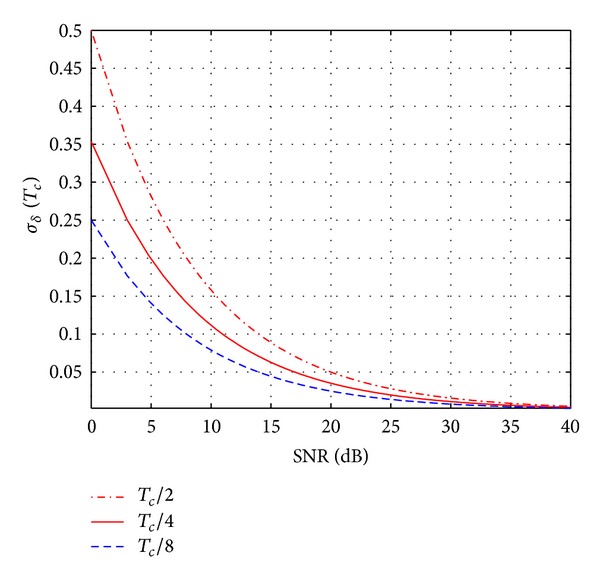
Tracking performance of the DLL versus SNR.

**Figure 7:**
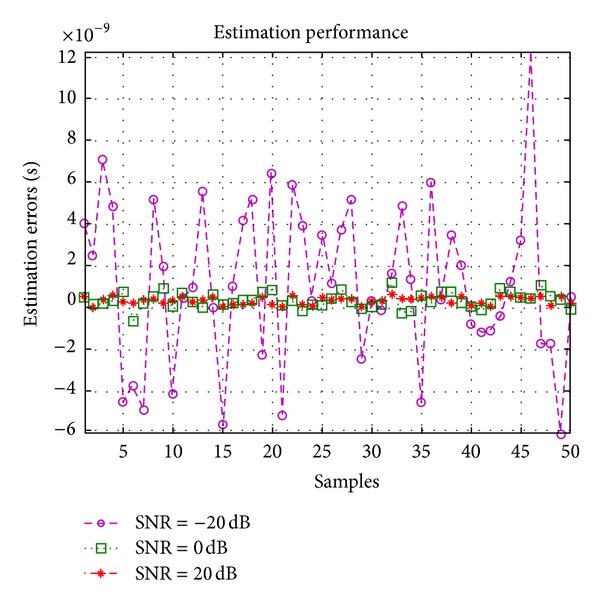
Statistical performance of the baseline estimation errors.

 An important performance criterion for DLL ranging estimation is the tracking jitter, that is, the variation of the delay error around the origin due to input noise, and this will ultimately affect the ranging accuracy. Consider the discriminator characteristics of the DLL as shown in Figure 5; it can be noticed that smaller delay offset gives a higher accuracy but a slightly smaller threshold acquisition range and a substantially smaller quasilinear region. There is another advantage of a smaller early-late gate delay spacing Δ_*ξ*_. For example, if Δ_*ξ*_ = *T*_*c*_/2 (*T*_*c*_ is one chip width of the PN codes), the squared autocorrelation is *R* ^2^(*T*_*c*_/2) = 1/4, whereas, for Δ_*ξ*_ = *T*_*c*_/8, it increases to *R* ^2^(*T*_*c*_/2) = 0.766, a 5.4 dB improvement. Note also that there are certain disadvantages if the early-late spacing becomes too small such as a slightly smaller threshold acquisition range and a substantially smaller quasilinear region. As a good compromise, Δ_*ξ*_ = *T*_*c*_/4 is used in the following simulations. Under the assumption that early-late gate behaves like a linear filter and the error is in the linear tracking area, the tracking jitter can be evaluated by [27]

(26) $$\begin{array}{c} \sigma_{\delta }=T_{c}\sqrt{\frac{\Delta_{\xi }\omega_{L}}{2\cdot \text{SNR}}}, \end{array}$$

where *ω*_*L*_ is the loop filter bandwidth.  Figure 6 shows the tracking performance of the DLL versus SNR. From Figure 7 we can conclude that this method can achieve satisfied results.

## 5. Conclusion

In distributed aerospace platforms, the baseline between the transmitting platform and receiving platform and the orientation of platforms towards each other are the most important parameters, and they are crucial for ensuring the system performance. To adaptively resolve the baseline and orientation estimation between distributed platforms for high-precision applications, an improved pulsed duplex microwave ranging method is presented in this paper, which allows determining the spatial baseline and orientation of two spaceborne platforms to each other in real time. Simulation results show that satisfactory measurement accuracy can be obtained for the approach. This approach is novel in the sense that it cancels the effect of oscillator synchronization errors due to that separate oscillators are used in the TX and RX platforms. Note that the influence of Doppler effect is neglected in this paper; we are aware that this contribution might become significant when there is severe intersatellite acceleration between the platforms; however, we rather choose to maintain a general overview character of our approach.

## Figures and Tables

**Table 1:** Possible phase errors caused by ADC.

*D *	4	8	10	12	14	16
*e*_*A*_	3.13*E* − 2	2.0*E* − 3	4.9*E* − 3	1.2*E* − 3	3.1*E* − 5	7.6*E* − 6
*δφ*	3.8°	0.22°	0.056°	0.014°	0.0035°	0.00087°
